# Digital Storytelling Methods to Empower Young Black Adults in COVID-19 Vaccination Decision-Making: Feasibility Study and Demonstration

**DOI:** 10.2196/38070

**Published:** 2022-09-26

**Authors:** Allysha Maragh-Bass, Maria Leonora Comello, Elizabeth Ellen Tolley, Darrell Stevens Jr, Jade Wilson, Christina Toval, Henna Budhwani, Lisa Hightow-Weidman

**Affiliations:** 1 Behavioral, Epidemiological, Clinical Sciences Division FHI 360 Durham, NC United States; 2 Duke Global Health Institute Duke University Durham, NC United States; 3 Hussman School of Journalism and Media University of North Carolina Chapel Hill, NC United States; 4 Gillings School of Public Health University of North Carolina Chapel Hill, NC United States; 5 DesignLab FHI 360 Durham, NC United States; 6 Institute for Global Health and Infectious Diseases University of North Carolina Chapel Hill, NC United States; 7 School of Public Health University of Alabama at Birmingham Birmingham, AL United States

**Keywords:** young Black adults, COVID-19, vaccine hesitancy, digital storytelling, community-based participatory research, digital health intervention

## Abstract

**Background:**

Despite high rates of novel COVID-19, acceptance of COVID-19 vaccination is low among Black adults. In response, we developed a digital health intervention (Tough Talks-COVID) that includes digital stories created in a workshop we held with young Black adults.

**Objective:**

Our formative research using digital storytelling workshops asked 3 research questions: (1) What issues did participants have in conceptualizing their stories, and what themes emerged from the stories they created? (2) What issues did participants have related to production techniques, and which techniques were utilized in stories? and (3) Overall, how did participants evaluate their workshop experience?

**Methods:**

Participants were workshop-eligible if they were vaccine-accepting based on a baseline survey fielded in late 2021. Final participants (N=11) completed a consent process, all 3 workshops, and a media release form for their digital story. The first 2 workshops provided background information and hands-on digital storytelling skills from pre- to postproduction. The third workshop served as a screening and feedback session for participants’ final videos. Qualitative and quantitative feedback elements were incorporated into all 3 sessions.

**Results:**

Digital stories addressed one or more of 4 broad themes: (1) COVID-19 vulnerability, (2) community connections, (3) addressing vaccine hesitancy, and (4) countering vaccine misinformation. Participants incorporated an array of technical approaches, including unique creative elements such as cartoon images and instant messaging tools to convey social interactions around COVID-19 decision-making. Most (9/11, 82%) strongly agreed the digital storytelling workshops were delivered as expected; 10 of 11 agreed (n=5) or strongly agreed (n=5) that they had some ideas about what story to tell by the end of the first workshop, and most (8/11, 73%) strongly agreed they had narrowed down their ideas by workshop two. Of the participants, 9 felt they would very likely (n=6) or likely (n=3) use digital storytelling techniques for personal use in the future, and even more were very likely (n=7) to use the techniques for professional use.

**Conclusions:**

Our study is one of the first to incorporate digital storytelling as a central component to a digital health intervention and the only one to do so with exclusive focus on young Black adults. Our emphasis on digital storytelling was shown to be highly acceptable. Similar approaches, including careful consideration of the ethical challenges of community-based participatory approaches, are applicable to other populations experiencing both COVID-19 inequities and marginalization, such as other age demographics and people of color.

## Introduction

### Background

The inequitable impact of the novel COVID-19 pandemic on Black, Indigenous, and other People of Color (BIPOC) communities has manifested itself in myriad ways. Beyond disparities in morbidity and mortality [1,2], the effects have included stigma, discrimination, and distress [3], particularly among those with multiple minority identities [4]. Inequities persist due, in part, to factors like frontline worker positions, higher rates of underlying comorbidities, and limited access to and quality of care [5]. All these challenges predated COVID-19, and in the era of telehealth, the digital divide has exacerbated them [6].

Despite greater likelihood of COVID-19 infection, Black communities have been slower to accept vaccination, due to longstanding historical misrepresentation, exclusion, discrimination, and exploitation in clinical research [7-9]. Furthermore, messages promoting the vaccine—through social and mainstream media outlets—reflect the voices of communities other than their own. Misinformation about COVID-19 vaccination, therefore, has spread widely, and lower rates of acceptance of COVID-19 vaccination among young adults are being seen [10]. Young Black adults, as frequent users of social media [11], have potential to play a crucial role in slowing the spread of misinformation and changing the narrative about the virus. Although they may be exposed to more potential sources of (mis)information, they also have potential to create content that is credible, engaging, and expressive of cultural pride [12].

To address these disparities, our team has developed a digital health intervention (Tough Talks: COVID) with a digital storytelling component featuring short videos created by and for young Black adults. Grounded in real-life experiences, these videos showcase how people have made decisions to get the vaccine and its impact on their lives. We describe digital storytelling workshops in which participants learned skills to create these videos, and we summarize the themes and features of videos, participants’ feedback, and personal takeaways. Therefore, this research is formative and documents the process by which digital storytelling approaches were used to empower young Black adults in COVID-19 vaccine decision-making. This work bridges disciplines of digital storytelling and community-based participatory research (CBPR); to our knowledge, it is the only work to do so in the context of vaccine uptake among young Black adults.

### Digital Storytelling and Community-Based Participatory Approaches

We define a story as a representation of thoughts or events with an explicit or implicit message about a topic, in line with other work on health narratives [13]. *Digital* stories often combine multimedia components (eg, video, audio) and are typically shared with wider networks online; the approach is well-suited to the experiences of communities experiencing marginalization due to factors like medical racism [14]. Although digital storytelling is relatively new, use of narratives has a long history in health behavior change [15]. Stories are especially effective when change requires overcoming resistance, processing complex information, and dealing with existential issues [13]—challenges that are all present in the context of preventing the spread of COVID-19 in Black communities. Stories may suspend habitual ways of thinking by eliciting emotion, enjoyment, or identification with characters and, in so doing, generate openness to new perspectives [16]. In the case of digital stories, the social validation of stories that are spread through social media may make them even more compelling.

Our project combines digital storytelling with the well-established approach of CBPR for addressing health inequities [17,18]. A hallmark of CBPR is involvement of stakeholders as partners in the research at all stages of the process, and in response, we engaged young adults as youth advisors to the project and as the creators of digital stories in our intervention itself. CBPR and digital storytelling are complementary approaches because both center the voices of communities, particularly those who experience marginalization. It is an ethical imperative to uplift the voices of these communities, and digital storytelling in the context of CBPR is an innovative approach in line with this. Further, the asset-building emphasis of CBPR [19] is congruent with our goal in digital storytelling workshops of providing skills training and support for digital storytelling. Despite these strengths, inherent to CBPR are several ethical considerations, including how to achieve a true community-driven agenda, how to navigate the tension of insiders versus outsiders, recognizing the limits of participation, and deciding on shared ownership and use of findings [20]. We are mindful of these issues and communicate regularly with our partners on them; in the discussion, we return to these considerations in light of our digital storytelling findings.

### Research Questions

With the goal of empowering young Black adults with the skills to create digital stories about their experiences with COVID-19 and vaccination, we conducted a series of workshops with 2 cohorts. We asked the following research questions (RQs):

- RQ1: What issues did participants have as they conceptualized their stories, and what themes emerged from the stories they created?
- RQ2: What issues did participants have related to production techniques, and which techniques were utilized in stories?
- RQ3: Overall, how did participants evaluate their workshop experience?

## Methods

### Overview

Digital storytelling workshops were conducted as part of a larger study (Tough Talks: COVID) aimed at testing a digital health intervention to empower young Black people in the southern United States to make autonomous decisions about COVID-19 vaccine receipt with culturally tailored and appropriate resources. The entire study (including digital storytelling activities) is guided by a youth advisory board comprised entirely of young Black adults in the 3 regions of the South where the study is being conducted (Alabama [AL], Georgia [GA], and North Carolina [NC]) and an expert advisory board comprised predominantly of Black experts in bioethics, intersectional stigma, health communication, faith-based initiatives, and CBPR. The aims of the larger study are to (1) conduct formative research to elicit the behavioral, cognitive, and environmental determinants influencing COVID-19 vaccine hesitancy among young Black people; (2) utilize implementation science to iteratively develop and refine the Tough Talks-COVID digital intervention with guidance from advisors; and (3) conduct a hybrid type 1 effectiveness implementation trial with young Black people from AL, GA, and NC to assess acceptability, feasibility, and effectiveness of the Tough Talks-COVID intervention on increasing COVID-19 vaccine uptake.

This research addresses the formative research proposed in Aim 1, which includes a series of digital storytelling workshops with young Black adults to create their own digital stories with potential to be included in the Tough Talks-COVID digital health intervention.

### Participants

Digital storytelling workshop participants were recruited through an online survey fielded in the larger study (N=150) from August 2021 to November 2021 and described elsewhere [21]. Respondents all self-identified as Black in survey demographic questions in the larger study from which workshop participants were identified. Median age was 23 (IQR 20-26) years. Over 80% (122/150, 81.3%) were vaccinated (1 dose of a 2-dose vaccine or fully vaccinated). Compared with cisgender women, cisgender men represented 27.3% (41/150) of the overall sample but roughly 40% (11/28, 39%) of unvaccinated individuals. Most participants had some college education or greater (125/150, 83.3%), and among essential workers (N=49), the proportion was roughly 30% (8/49, 16%). Participants were eligible for workshops if they reported willingness to or previous completion of a COVID-19 vaccine series in the survey. Each survey respondent who was eligible and expressed interest was scheduled for a meeting with a study team coordinator to be consented and address any questions about participation. Our final workshop participants (N=11) were those who completed the survey and all workshop activities. Participants were divided into 2 cohorts to (1) offer 2 sets of dates for which participants could gauge their availability and (2) ensure a small enough group size that all participants were given ample time to work in small groups with our study team when developing their digital stories. With our final group, cohort 1 had 6 participants, and cohort 2 had 5 participants.

### Digital Storytelling Workshops

All workshops took place on a Health Insurance Portability and Accountability Act (HIPAA)–compliant Zoom platform and were scheduled on weekday evenings accommodating work and school schedules. Each workshop lasted between 120 minutes and 150 minutes and were held 2 weeks apart from October 2021 to November 2021. Workshops were led by a group of 5 facilitators, 4 of whom identify as people of color, and 3 of whom identify as young Black adults.

The goal of the series was to help participants develop a digital story (1 minute to 3 minutes) for use in the Tough Talks-COVID intervention. The series was designed to be comprehensive yet approachable to participants with varying levels of experience with digital storytelling. The first 2 workshops provided background information and hands-on digital storytelling skills from pre- to postproduction. The third workshop was a screening and feedback session for participants’ final videos, as well as an opportunity to discuss how participants could highlight their digital storytelling workshop experience on their resume and professional lives. Additionally, the third workshop combined participants in both cohorts so that they could serve as audiences for each other’s videos. Most but not all participants in our 2 digital storytelling cohorts chose to screen their videos and discuss their experiences during a final “showcase” event attended by community partners and the wider research team.

**Figure 1:**
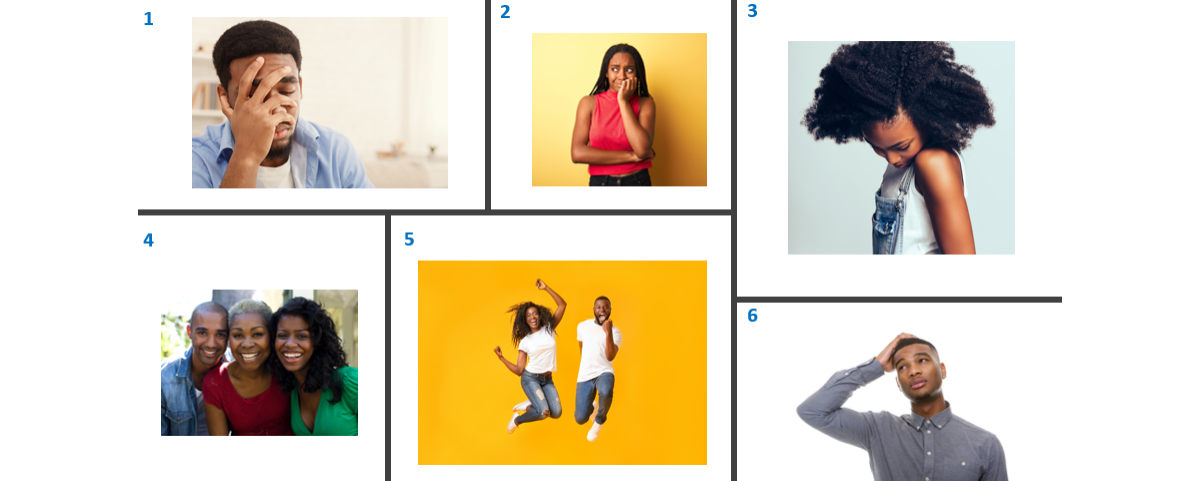
Emotion card.

Because of the virtual format of the sessions, it was important to keep participants engaged. One strategy was the use of ice breakers, such as asking a participant to name their city of birth and favorite food and to “popcorn” it to another participant to do the same until everyone had shared. Another ice breaker involved creating a collaborative story, in which a workshop facilitator began a story with a one-sentence opening and passed it to another person who would then add another sentence, until all facilitators and participants had contributed. Another engagement strategy was the use of emotion cards to gauge participants’ comfort level with different activities (see Figure 1). Cards featured 6 licensed stock images of young adults of color and were numbered from 1 to 6, without additional labels but with clearly portrayed emotions (eg, shyness, excitement). In each case, we invited participants to say or type the number corresponding to the emotion and then, in their own words, describe what they would call the emotion. All sessions ended with a recap of key information and any reminders for the next session.

### Sources of Feedback

#### Qualitative (Brainstorming Discussions and Breakout Groups)

In addition to workshop engagement approaches, we incorporated qualitative feedback and evaluation measures into each workshop. During session one, we utilized Google Jamboards to facilitate discussion around 2 key questions: (1) What COVID-19 vaccine stories need to be told? and (2) What could these stories look and sound like? During session two, the same Jamboard questions were utilized but with more focus on the themes and technical aspects of the “practice” videos they were asked to create. Facilitators with digital storytelling expertise led breakouts to address any specific editing or technical questions. During session three, we utilized Jamboard to facilitate discussion related to 3 questions: (1) How do you feel about the workshops, now that you have completed them; (2) How would you explain the experience to others who may not be familiar with digital storytelling; and (3) How (if at all) could you use these skills moving forward? Team members recorded responses in real time to all questions in the form of “sticky notes” and actively confirmed with participants while writing them that the notes were accurately representing their feedback. Thematic analyses were conducted by team member EET and reviewed and discussed with team members AMB, MLC, and DS to ensure they were grounded in feedback from participants.

#### Quantitative (Feedback Forms)

At the completion of session three, participants completed a 9-question feedback form about their experience. Each question utilized a 4-point Likert scale and included items such as “The digital storytelling workshops were like what was explained to me during the consent process,” “How likely are you to use digital storytelling in the future for your professional use?” and “After completing the digital storytelling workshops, I feel this was worth my time.” Descriptive statistics were conducted with the total number of participants who completed all 3 workshops (N=11). Between sessions one and two, 3 participants from the original 14 discontinued participation due to work schedules or inability to complete a digital story in time.

### Ethical Considerations

All workshop participants completed (1) a survey, (2) an informed consent discussion with our study coordinator prior to consenting to the workshops with opportunities to ask questions, (3) all 3 workshop sessions, and (4) a media release form consenting to the use of their digital story in the Tough Talks-COVID digital health intervention in addition to a consent form for participation in the workshops. For all sessions, participants were asked to keep cameras on, if preferred, and to share names and pronouns; participants were also able to use preferred names or pseudonyms in all sessions. Participants received US $50 for each session in which they participated and an additional US $75 for successful video creation. All digital storytelling workshop activities were fully approved by the University of North Carolina at Chapel Hill Institutional Review Board (IRB# IGHID 12112).

## Results

Across the 2 cohorts of participants in the workshop series, 11 unique videos were created, ranging in content and technical approach and from 1 minute to 3 minutes in length. In the following sections, we detail the workshop sessions and final videos, organized by our 3 research questions on the types of stories told (RQ1), the video and digital techniques used (RQ2), and participants’ overall assessment of the process (RQ3.)

### RQ 1: Issues Related to Storytelling and Types of Stories Told

**Figure 2:**
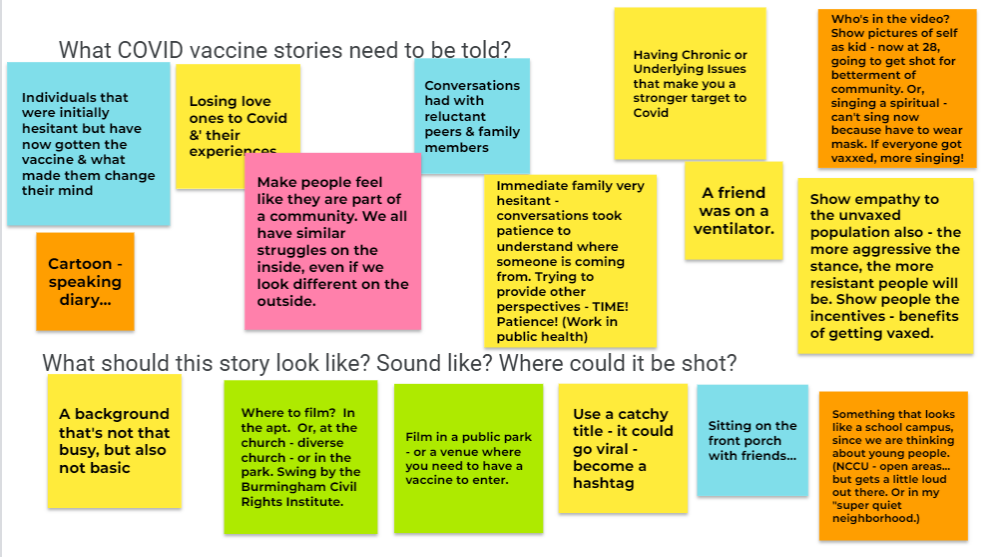
Digital story content brainstorming and development in workshop cohort #1. NCCU: North Carolina Central University.

**Figure 3:**
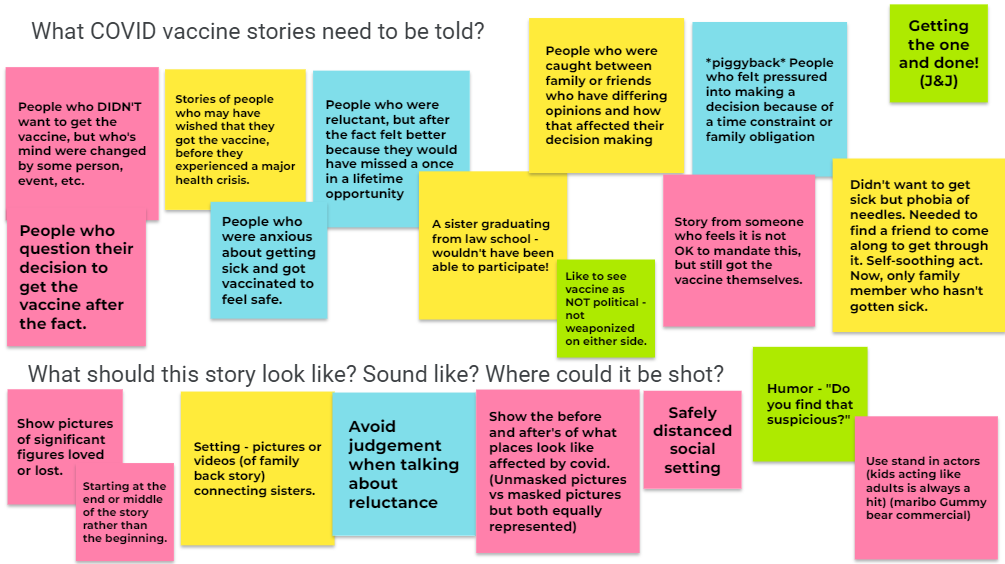
Digital story content brainstorming and development with workshop cohort #2.

Although some participants were initially anxious about determining what story “should be told,” the digital storytelling process helped most to develop their own unique story about COVID-19 and to answer the question “Why get the vaccine?” Summarized in the following sections and in Figures 2 and 3 are sample “sticky notes” from participant brainstorming in sessions one and two prior to the final digital stories’ creation.

Our summary, therefore, also describes the content ultimately included in videos, which centered around one or more of 4 broad themes, which we have labeled (1) COVID-19 vulnerability, (2) community connections, (3) addressing vaccine hesitancy, and (4) countering vaccine misinformation.

#### COVID-19 Vulnerabilities

As participants worked through initial story ideas in digital storytelling workshop sessions one and two, fear of getting COVID-19 was a common theme. Participants suggested that fear (of getting COVID, of losing a loved one to the disease, or of concerns that chronic underlying conditions could make someone more vulnerable to COVID-19 death or long-term health effects) was a strong motivating factor for vaccine uptake. Within this theme, a range of stories emerged, including a first-person experience of having gotten COVID-19 and the emotional and physical effects experienced; a story about a friend who was on a ventilator; and a story about a COVID-19 naysayer who regretted not having gotten the vaccine after suffering a major health event (see Figures 2 and 3).

#### Community Connections

A second theme was more positive framing focused on family and community connections. Participants emphasized the need to make people feel like part of a community. They offered images of, for example, gatherings among extended family and singing spirituals together. One approach to conveying this theme was to highlight that we all face similar struggles and that getting the vaccination shows concern about and for the community. A second focused on the idea of a return to normalcy and being able to live one’s best life. For example, getting vaccinated meant that a person could move back out into the world, connect with friends and family members, and participate once again in important events, such as seeing a sister graduate from law school.

#### Addressing Vaccine Hesitancy

Several participants acknowledged that discussing COVID-19 vaccines was challenging, either for themselves or for their families. One participant indicated that people had strong opinions on both sides—both for and against the vaccine. She and others intimated that the vaccine should not be politicized or weaponized on either side and that the more aggressive the stance, the more resistant people will be. To be most effective, we must show patience, provide different perspectives, and be respectful in conversations with reluctant family members. Several ideas emerged during brainstorming, including showing someone who did not want the vaccine but whose mind was changed by an event or person, someone who feels it is not OK to mandate but still got the vaccine themselves, or someone afraid of needles but who was helped by a friend to get through it.

#### Countering Vaccine Misinformation

A final theme focused on the need to debunk vaccine rumors and misinformation. Participants noted that some members of their community raised questions about what is in the vaccine and whether the vaccine could change your DNA. They also noted deep-seated mistrust of the medical establishment and concerns that previous vaccines had been tested on Black people as well as lingering resistance because you can still get COVID-19 if vaccinated. Final videos reflected these concerns, as well as various counterarguments participants offered. For example, one participant equated motivations for prevention of COVID-19 and pregnancy by pointing out that we still encourage condom use even though “you can still get pregnant.” Some highlighted the persuasiveness of using celebrities to discuss and combat vaccine misinformation or of featuring positive experiences from breastfeeding moms who got the vaccine.

### RQ 2: Issues Related to Production Techniques and Techniques Used

#### Production Technique Issues and Process

In the first 2 workshops, our video experts reviewed basic technical procedures and how-to tips on filming and editing videos. Digital storytelling participants were invited to consider several technical aspects as they planned their videos. They included (1) venues, (2) how to shoot their video, (3) who the “speaker” for the video is, and (4) any unique creative elements they might use.

Regarding brainstorming venues, participants seemed comfortable envisioning appropriate backdrops. In the breakout sessions, participants suggested a diverse set of backgrounds, including a public park or church, the Birmingham Civil Rights Institute, or sitting on a porch with friends.

Regarding how to record videos, during breakout sessions, a few participants described previous experience shooting and editing videos, but most had not had much experience with that and expressed concern about being able to produce videos. Our video experts reminded them to be gentle with themselves, shoot more, and edit out. Participants were also encouraged to use the technology they had (cell phone, tablet, or computer) rather than obtaining special equipment or software.

Regarding speakers, as they brainstormed initial story ideas, some participants seemed comfortable placing themselves at the center of their stories, while others imagined their story delivered by others (for example, a trusted elder or stand-in actors).

For unique creative elements, ideas generated during the breakout sessions included music or dramatic news headlines to set a somber tone and then shifting scenery, speaker, or music to move viewers’ emotions from dark or anxious to hopeful. Other ideas included depiction of an instant messaging conversation between friends who were supporting each other to resist vaccine-related misinformation.

#### Technical Approaches Used in Final Digital Stories

Summarized in Table 1 are each of the 11 videos. Most of the videos (n=7) incorporated music at some point during the story to convey emotions or to serve as a backdrop. Only a handful of stories utilized subtitles (n=3), most notably used in a video that depicted a text message–based conversation with peers about COVID-19 vaccines. Unique creative elements were seen in every digital story, which shows the individual creativity of each creator using the skills taught during the workshops. The elements included, but were not limited to, continuous narration (video 1), animations to add visual interest (videos 2 and 8), multimedia platform usage with transitions (video 4), and incorporation of news or social media headlines and addressing misinformation (videos 9 and 11).

**Table 1:** Digital story loglines and content from digital storytelling workshop participants (N=11).

Video	Logline	Sex	Age (years)	Theme(s)	Music	Subtitles	Unique creative elements	
1	A young student somberly reflects on why she chose to get the COVID-19 vaccine.	F^a^	21	Community connections Addressing vaccine hesitancy	Yes	No	The video is narrated with overlayed audio and edited together with simple transitions. There is a good balance of narrative footage (where she speaks directly into the camera) and cutaway footage (contextual video clips), which provide a clear digital story. Ambient music played softly in the background to strengthen the reflective tone of the video.	
2	When a young woman is not receiving support from her family about the COVID-19 vaccine, her close friends are there to give her the validation she needs.	F	21	COVID-19 vulnerability Community connections Countering vaccine misinformation	No	Yes	The video begins with a short title and introductory slide, then quickly fades to a simulated screen-capture of someone typing and sending a text message (recreated via animation). The video features 2 text message threads. The first conversation is between her and some unsupportive family members, and the second is between her and a group of supportive friends. Each is clearly demarcated with different message colors, and the conversations are separated by a narrative slide with bold yellow text on a black background. The video ends with a closing slide that is styled in the same way for visual continuity. The video effectively uses sound effects to replicate the experience of an active text conversation.	
3	When a young woman is hesitant about receiving the COVID-19 vaccine, the possibility of missing out on major family moments motivates her to decide.	F	26	COVID-19 vulnerability Community connections Addressing vaccine hesitancy	Yes	Yes	The video begins with a blank screen and the sounds of a person getting into their car/turning on the radio. Footage from driving to a pharmacy is shot from the car dashboard (street view). It also captures her walking into the pharmacy for her vaccination appointment. This “in-car” perspective shows up again in footage of her family celebrating her sister’s graduation from their car. Throughout the video, black slides with white text fade onto the screen to move the narrative along. Although the first part of her video does not include music (just natural background sounds from her car), in the second half of the video, she overlays upbeat music on a slideshow of pictures and video clips capturing the postgraduation festivities. The music contrast conveys the contrast of her initial feelings of anxiety and hesitancy with her eventual excitement and relief.	
4	A young woman experiences a date-gone-wrong when talking about COVID-19 misinformation with her date.	F	24	Community connections Countering vaccine misinformation	No	No	The video begins with a Zoom-esque call between her and her 2 friends. All 3 videos are displayed on the screen (gallery style), and you can see that they are filming from different locations. They greet each other, and her friends are curious to know more about a date she recently went on. After sharing that it did not go well, she starts to describe the last moments of the date. She begins, “*Pretty much it went like this…*”—the video uses a jump cut to take us directly to a scene/conversation from her date. The dialogue scenes are edited together using a mix of wide and closeup shots. The wide-shot framed her and her date as they sit on a couch. Tighter, over-the-shoulder shots focused in on her date and his perspective on COVID-19. The date ends with her wishing him well and encouraging him to change his views on COVID-19 vaccination. The video jumps back to the video call between the sisters for the final wrap up. They laugh together and end their video call.	
5	A young mother reflects on her most isolating and impactful moments from the beginning of the pandemic.	F	26	COVID-19 vulnerability Community connections	Yes	No	The video begins with dramatic music and an all-caps title slide that reads “2020 THE YEAR OF COVID-19.” The words are bright red and are on a black background. As the burst of music fades, the video quickly transitions to footage of her speaking directly to the camera. She filmed outdoors, her lighting was even, and the audio was clear.	
6	A young woman wants to encourage people to get the COVID-19 vaccine with hopes of getting our lives back before the pandemic.	F	23	COVID-19 vulnerability Community connections	Yes	No	The video opens with a title slide and fades to footage of a woman sitting on a sofa—staring straight ahead. As the strings play the intro of a well-known, somber, soul music song, the video zooms in on her emotionless expression. The clip has a blue monotone filter, and the year 2020 is fixed to the top left corner of the screen. As the camera inches closer, she stretches out her TV remote to signal that she is changing the channel as the music plays for a few bars. The screen transitions to black and uses a quick *iris close* transition to mimic turning off a TV screen. At the same time, she uses the *turntable rewind* sound effect to signal a shift in the narrative. The screen is black, and the date 2021 appears in the top left corner. An uplifting, high-tempo, R&B song begins playing. As the music builds, you see footage of her entering a pharmacy for her vaccine appointment. The visual style of the video changes from this point on, showing full color and the edits timed to match the upbeat rhythm of the song. In some areas, she plays with speeding up the footage or removing frames (stop-motion aesthetic) to highlight special moments (eg, showing off her vaccination Band-Aid, hugging family). The video continues in this style as she drives to her family’s home to play Monopoly. As the footage continues and the music plays softly, she adds a voiceover narration. She encourages people to get vaccinated so we can “safely get back to enjoying life.” The video ends with an end card slide and #ToughTalks.	
7	A classroom engages in insightful conversation on the importance of the COVID-19 vaccine.	F	26	Community connections Addressing vaccine hesitancy Countering vaccine misinformation	No	No	The video begins with a stock photo of a smiling teacher standing in front of a chalkboard. The video continues in this aesthetic and combines voiceovers with stock photos to visualize a conversation between a teacher and inquisitive students. The transitions cut from photo to photo, and the photos correspond with the character voice that is speaking (characters: teacher and students Ashely, David, Veronica, Lindsey).	
8	COVID-19 superwoman shares tips and information about the COVID-19 vaccine.	F	19	COVID-19 vulnerability Community connections	Yes	No	The video is informational and presented as a series of animated slides. Some of the slide graphics include simple animations to add visual interest. The music is ambient and does not distract from the content.	
9	A young woman addresses myths about the COVID-19 vaccine.	F	19	Addressing vaccine hesitancy Countering vaccine misinformation	Yes	No	The video begins with dramatic, high-energy music playing in the background and a screenshot of a tweet from a well-known rapper who has expressed COVID-19 vaccine hesitancy through social media. After a few seconds, the clip and music abruptly stop, and the video cuts to a CNN news clip addressing the misinformation in the rapper’s tweet. With the context and format established (present misinformation, and address it head on), her video transitions to her speaking directly to the camera. She continues to address common rumors and misinformation surrounding COVID-19. She used graphics and supporting screenshots as she told the story.	
10	A young woman reflects on her experience with missing loved ones and how getting vaccinated brought back the connections she longed for.	F	24	COVID-19 vulnerability Community connections	No	No	The video is a personal account of a young woman’s experience with COVID-19. The video was filmed in one continuous take, and she shared her story by speaking directly into the camera. Her background was styled with neutral décor to add visual interest but not distract from herself. Her lighting was in front of her and even cast her face, and her audio was consistent and clear.	
11	A young woman shares how her mother being a nurse influenced her decision to get the COVID-19 vaccine.	F	22	COVID-19 vulnerability Community connections	Yes	Yes	The video is set in New York. It opens with a slide that reads “My COVID-19 experience was so much different than anything I’ve heard” as a somber, hopeful piano track plays in the background. The video transitions between news clip headlines, photos of empty city streets, and exhausted health care workers. While the music continues in the background, a news clip featuring an interview with her mother, a nurse, plays. Throughout the video, black slides with white text fade onto the screen to move the narrative along.	

^a^F: female.

### RQ 3: Overall Assessment of the Digital Storytelling Workshop Process

**Figure 4:**
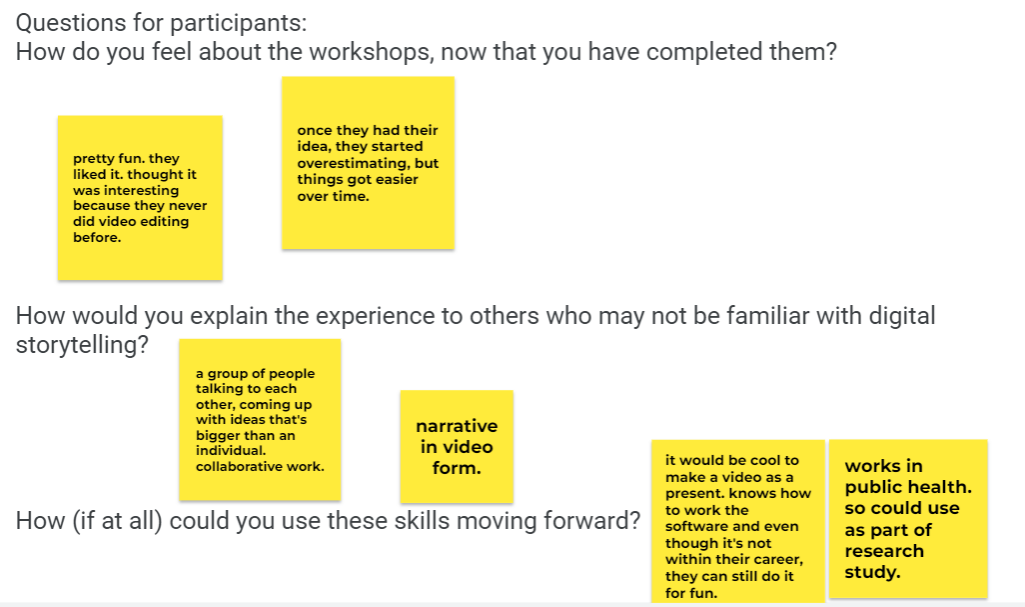
Digital storytelling workshop feedback from participants during the final session.

In the quantitative workshop feedback, participants identified both professional and personal value from learning how to tell digital stories. Based on evaluation forms and informal feedback at the end of the third workshop, participants appreciated learning technical skills for both storytelling and videography (Figure 4).

All participants agreed that their time was well spent, with almost three-quarters (8/11, 73%) strongly agreeing. The majority (9/11, 82%) strongly agreed that the digital storytelling workshops were like what had been explained to them; 10 of 11 agreed (n=5) or strongly agreed (n=5) that they had some ideas about what story to tell by the end of the first workshops, and most (8/11, 73%) strongly agreed they had narrowed down their ideas by workshop two. Of the 11 participants, 9 felt they would very likely (n=6) or likely (n=3) use these digital storytelling techniques for personal use in the future, and even more were very likely (n=7) to use the techniques for professional use. Most participants (6/11, 55%) spent between 3 hours to 4 hours shooting their videos. Just under one-half (4/11, 36%) spent about 3 hours to 4 hours editing their video. All participants said they would be interested in being contacted in the future for a related Tough-Talks: COVID or other research activity.

When asked about how they might explain what they learned about digital storytelling to others, one participant described it as “sort of like TikTok but with really personal stories,” while another described it as learning “how to put your ideas and experiences out there for others—not just for your family.” One participant liked learning how to frame ideas more clearly and script them to be purposeful. Several participants appreciated the introduction to video editing and acknowledged that these (new-found, for many) digital storytelling skills could help with classes, presentations, job searches, and work-related activities. Participants came to these workshops with different motivations and expectations. Several joined out of curiosity or to accept a challenge; some worried that their personal story would not measure up. All who completed the workshop series shared positive remarks at the end. On the personal side, one participant reflected that she now felt prepared to share not just digital stories but also personal stories. Many of the participants felt validated or even empowered from hearing the diverse perspectives about why their peers had chosen to get vaccinated.

## Discussion

The purpose of this research was to describe the use of digital storytelling methods to empower young Black adults in the South who continue to experience COVID-19 disparities by documenting what type of stories were told and how they emerged, as well as the way in which these individuals felt about the process.

### Principal Findings and Application to Ongoing Study Activities

Overall, we observed high satisfaction with workshop participation, and every participant reported professional benefits to digital storytelling training and that workshops were a good use of their time. This is a unique innovation of our approach, which, when compared with PhotoVoice, may offer more professional as well as personal relevance to young people who often share about their lives in social media formats but may not be empowered to describe their views on COVID-19 vaccine decision-making.

The videos produced have potential to resonate with peers because they reflect the unique voices of participants and may invoke shared experiences. Videos also addressed risk factors for Black communities (such as vaccine misinformation) and highlighted cultural strengths (such as community connections). Stories were conveyed using a variety of techniques, most of which were more sophisticated than simply capturing video on one’s phone.

In future digital storytelling workshops, our team would increase the interactive nature of the sessions, such as a devoted workshop session where draft versions receive hands-on guidance from our content and technical team leaders. Another improvement for future workshops would be a brief inventory about familiarity with digital storytelling that could be shared with participants ahead of workshops to gauge their existing familiarity with and interest in social media engagement and digital storytelling. This type of inventory would be adapted to other health topics and also other populations to better tailor workshop content. Lastly, given the engagement of our youth advisors throughout all aspects of the study, youth advisors could be invited to attend digital storytelling workshops, create digital stories themselves, or lead aspects of the training that may yield professional benefit to them like that expressed by workshop participants.

### Comparison With Prior Work

Our approach, guided by CBPR principles, was conducive to centering the needs and voices of communities experiencing marginalization, such as young adults of color. Compared with common CBPR methods such as PhotoVoice, digital stories similarly focus on empowering communities, with added innovative ability to incorporate mixed media and conduciveness to dissemination through social media. Given the ubiquity of social media, digital storytelling holds powerful potential to combat COVID-19 misinformation when told from the vantage point of young Black adults to peers.

As the larger Tough Talks-COVID study gets underway, we continue to consider ways to address the ethical standards of CBPR described earlier: (1) how to achieve a true community-driven agenda, (2) the tension of insiders versus outsiders, (3) the reality of participation limitations, and (4) shared ownership and dissemination of findings to drive action [20]. We have worked to achieve a true community-driven agenda with the formation and maintenance of youth and expert advisory board members who are from and work in the communities in the South where our study is based. Our experts include community advocates, bioethicists, pastors, and vaccine scientists and is 93% Black (youth advisory board is 100% Black). To date, our advisory board members have edited and tested our baseline surveys, informed the scripts for informational videos that are being built into the Tough Talks-COVID intervention, and were featured judges for a showcase where our digital storytelling workshop participants competed for additional cash prizes from incentives previously mentioned.

Next, we navigate insider-outsider tension by having several team members who identify as young people of color (3 of the 5 digital storytelling team members), and most of our larger Tough Talks-COVID team live and work in the 3 regions where the study is being conducted with a longstanding history of community-engaged work. Third, while digital storytelling workshop participants responded positively to engagement methods such as the audience-specific emotion cards, future study activities can do more engaged work via in-person activities that were not feasible for us considering the COVID-19 global pandemic. Lastly, shared ownership and dissemination include that, upon progression of the study, participants will be invited to share their digital stories on their own social media. We will also recontact workshop participants, since all consented to further study contact, to invite them along with youth advisors to conduct their own content analysis of their digital stories and provide training to them on this along with how to craft a scholarly manuscript that they will coauthor.

### Limitations

The present findings are subject to limitations. Our digital storytelling workshops (and the broader Tough Talks-COVID study) focus on young Black adults in the South and was limited in size. Although there are theoretical, epidemiological, and empirical reasons for doing so, the exclusive focus may limit the generalizability of our findings. We found that participants who self-selected into workshops were 100% vaccine-accepting despite our invitation being open also to those who are vaccine-ambivalent. Greater variability may have yielded different stories and themes with greater potential to be useful to the Tough Talks-COVID app. The videos produced had little male representation, reflecting the demographics of the larger survey sample from which the participants were selected. Additionally, 2 men dropped out before completing the workshops. Our workshops did not emphasize diversity in ethnicity (eg, Hispanic/Latinx, Afro-Caribbean), sexual orientation and gender identity, or disability status. Finally, although we describe themes and features of stories and participants’ perceptions of workshop experiences, these findings do not speak to the effectiveness of videos, individually, on influencing attitudes and behaviors or in combatting misinformation. Experimental research is needed to assess that kind of impact. Selected videos will be assessed as a component in a larger digital health intervention by the Tough Talks-COVID study.

### Conclusions

Despite limitations, to our knowledge, our Tough Talks-COVID study is one of the first to incorporate digital storytelling as a central component to a digital health intervention and the only one to do so with exclusive focus on young Black adults. We feel this is appropriate, given the deeply rooted mistrust of many medical establishments including those who have produced the COVID-19 vaccine, which has often taken the form of misinformation spreading among young adults, including through social media. Our emphasis on digital storytelling was shown to be highly acceptable, and future activities with this approach in our study will engage more diversity within this population to contribute additional digital stories and invite young people to disseminate these stories on their own social media as a powerful peer-driven approach to combat COVID-19 misinformation online. Similar approaches, including careful consideration of the ethical challenges of CBPR approaches, are applicable to other populations experiencing both COVID-19 inequities and marginalization, such as other age demographics and people of color. More research is needed to disseminate this innovative approach and leverage its profound potential to uplift the voices and needs of communities experiencing marginalization and COVID-19 inequities.

## Abbreviations

BIPOC: Black, Indigenous, and other People of Color
CBPR: community-based participatory research
HIPAA: Health Insurance Portability and Accountability Act
RCT: randomized controlled trial
RQ: research question
